# A257 EARLY PROACTIVE THERAPEUTIC DRUG MONITORING WITH USTEKINUMAB THERAPY IN PAEDIATRIC CROHN’S DISEASE

**DOI:** 10.1093/jcag/gwad061.257

**Published:** 2024-02-14

**Authors:** A Ricciuto, H E McKay, J deBruyn, E Crowley, P Church, H Huynh, A Otley, A Shaikh, W El-Matary, E Wine, T Walters, A Griffiths

**Affiliations:** Paediatrics, The Hospital for Sick Children, Toronto, ON, Canada; Paediatrics, The Hospital for Sick Children, Toronto, ON, Canada; University of Calgary, Calgary, AB, Canada; London Health Sciences Centre, London, ON, Canada; Paediatrics, The Hospital for Sick Children, Toronto, ON, Canada; University of Alberta, Edmonton, AB, Canada; Dalhousie University, Halifax, NS, Canada; Paediatrics, The Hospital for Sick Children, Toronto, ON, Canada; University of Manitoba, Winnipeg, MB, Canada; University of Alberta, Edmonton, AB, Canada; Paediatrics, The Hospital for Sick Children, Toronto, ON, Canada; Paediatrics, The Hospital for Sick Children, Toronto, ON, Canada

## Abstract

**Background:**

Ustekinumab (UST) is an effective therapy for adults with moderate to severe Crohn’s disease (CD), but data concerning optimal dosing in children are sparse.

**Aims:**

To examine real-world post-induction PK and efficacy in a prospective multicentre cohort study of paediatric CD (Canadian Children IBD Network (CIDsCaNN)).

**Methods:**

Children 2-17 years-old with CD were eligible for this analysis if they received IV UST for treatment of luminal CD and had serum UST levels measured at week 8. Median with interquartile range (IQR) UST dose and serum trough levels at weeks 8 and 16 were compared between children ampersand:003C40 kg and ≥40 kg with Mann-Whitney U test. Clinical remission was defined as weighted Pediatric CD Activity Index (wPCDAI) ampersand:003C12.5. UST durability was compared between those with a week 8 serum UST level ampersand:003C5 versus ≥5 µg/mL (log-rank p-value).

**Results:**

Between 21/04/2017 and 30/04/2023, 39 children were eligible (74% M; median age 13.5 (IQR 11.9-15.6) y; median weight 44.7 (IQR 30.4-55.0) kg, 12 children ampersand:003C40 kg; 84% bio-naïve). Median disease duration at UST start was 4 (IQR 1-17) months; CD location distal ileal ± limited cecal (18%), colonic (26%), ileocolonic (54%). Median follow-up duration was 10.0 (IQR 6.7-12.7) months. All children received UST IV induction (67% 260 mg, 18% 390 mg) followed by 90 mg SC every 8 weeks. Induction doses in mg per kg were higher in those ampersand:003C40 kg (median 9.11 (IQR 8.68-10.08)) compared to those ≥40 kg (5.77 (IQR 5.24-6.45) mg/kg), pampersand:003C0.001. Induction doses in mg per body surface area (BSA) in m^2^ were similarly higher in those ampersand:003C40 kg (median 251.66 (IQR 244.16-258.99) versus 195.15 (IQR 172.71-224.11) mg/m^2^, pampersand:003C0.001). Despite higher induction doses, week 8 trough levels were numerically lower in those ampersand:003C40 kg (median 4.32 (IQR 1.33-8.36) versus 6.96 (IQR 5.38-8.90) µg/mL, p=0.29). Week 16 serum UST levels were also numerically lower in ampersand:003C40 kg versus ≥40 kg (median 4.04 (IQR 1.92-6.98) versus 5.50 (IQR 3.17-8.27) µg/mL, p=0.57). Interval shortening during maintenance to every 4 weeks occurred in 7 children prior to week 16, and in 14 children prior to 6 months. By 4 months, 19 of 33 children with available data (56%) achieved clinical remission. Overall, 8/39 (21%) ceased UST. Those who discontinued UST had significantly lower week 8 levels than those who continued UST (median 3.52 (IQR 1.19-6.12) versus 7.15 (IQR 5.09-10.15) µg/mL, p=0.027). In survival analysis, week 8 trough level ampersand:003C5 µg/mL was associated with an increased risk of UST discontinuation (log-rank p=0.032) (Figure 1).

**Conclusions:**

Higher UST concentration at week 8 is associated with greater drug durability. A higher BSA-based induction for children ampersand:003C40 kg is supported by higher serum drug levels and improved drug durability.

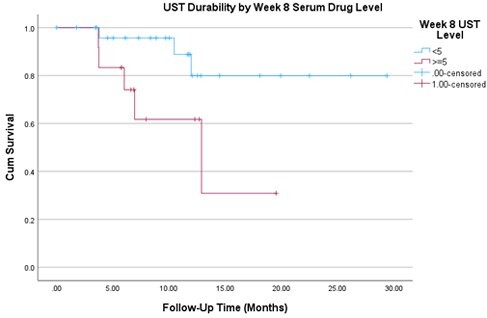

Figure 1. Ustekinumab durability by week 8 serum drug level

**Funding Agencies:**

CIHR

